# Surgical and hormonal gender-affirming care: cross-domain determinants and artificial intelligence-enabled expansion

**DOI:** 10.1186/s12939-026-02870-7

**Published:** 2026-05-04

**Authors:** Mehek Sharma, Vikram Sharma, Gianluca De Leo

**Affiliations:** 1 Medical College of Georgia at Augusta University, Augusta, GA 30912 USA; 2 Brown University, Providence, RI 02912 USA; 3 Department of Health Management, Economics and Policy, Augusta University, Augusta, GA 30912 USA

**Keywords:** Gender-affirming surgery, Gender-affirming hormone therapy, Transgender and gender-diverse populations, Health equity, Artificial intelligence in healthcare

## Abstract

Gender-affirming surgery (GAS) and gender-affirming hormone therapy (GAHT) and are evidence-based components of care that support the health and well-being of transgender and gender-diverse (TGD) individuals, with extensive evidence linking them to reduced suicidality, improved mental health, and quality of life. Whereas most studies examine isolated outcomes—often within urban populations—this review adopts a system-level, cross-domain perspective to examine how access to gender-affirming care is shaped by structural conditions, including the urban concentration of affirming services, limited clinician training, fragmented insurance coverage, and the underrepresentation of rural TGD populations in research. These intersecting barriers undermine timely initiation, continuity, and quality of GAHT and GAS across diverse care settings. A distinctive contribution of this review is its integration of artificial intelligence (AI) as an emerging dimension of gender-affirming care. By synthesizing evidence on AI-enabled decision support, training simulations, and voice therapy, the review positions AI as a promising, equity-enhancing pathway to expand access, strengthen provider competency, reduce administrative burden, and advance global TGD health equity at scale.

## Introduction

For transgender and gender-diverse (TGD) individuals worldwide, gender-affirming care is being increasingly recognized as essential for health, dignity, and survival [1]. Clinical evidence in support of interventions such as gender-affirming hormone therapy (GAHT), gender-affirming surgery (GAS), voice therapy, and psychosocial support has shown to reduce suicidality, improve mental health, and promote overall well-being [2]. However, access to this care remains one of the starkest inequities in medicine, limited by systemic, policy, and structural barriers [2]. This divergent reality exposes vast gaps in local health system readiness, geographic disparities, provider competence, and entrenched stigma [3]. The fragmented TGD care landscape leaves TGD populations vulnerable to inequities in health and social support [4]. This inequity becomes pronounced in rural and underserved environments where TGD people face delayed or unsafe care, higher mental health burdens, and increased exposure to violence [5]. Even when laws lag or recognition is denied, the need for gender-affirming care does not disappear [4]. The need for competent, affirming care is not a matter of convenience but a determinant of survival [6, 7].

Gender-affirming care at its core is about affirming human identity through medicine [7]. In recent years, global progress in the form of policy reforms, clinical guidelines, and community advocacy has improved the recognition of TGD health needs in many countries [7]. Specialized clinics have been established, legal protections enacted, and training programs piloted to expand access [8]. However, these gains remain highly uneven, with services disproportionately clustered in urban centers and resource-rich settings [3]. Studies from diverse regions including Asia, Europe, and North America reveal ongoing disparities shaped by age, socioeconomic position, insurance coverage, and local sociopolitical climates [9]. However, the barriers extend beyond travel distance to encompass cultural stigma, scarcity of affirming providers, and financial burdens associated with accessing care far from home [10]. Research highlights that rural TGD adults experience lower rates of hormone therapy initiation and longer waits for surgical procedures, despite comparable health needs [10]. In some cases, patients must cross state lines, or even national borders, to obtain basic services [9]. For many TGD people, these burdens exacerbate existing challenges from poverty, lack of insurance, or limited social support [5]. Rural TGD populations are also underrepresented in the literature, resulting in a knowledge gap about the scale of their unmet needs [9].

Medical provider preparedness is emerging as an overlooked determinant of TGD care [11]. Many healthcare professionals report little to no formal training in GAHT or GAS, leaving them uncertain about prescribing hormones, managing surgical referrals, or addressing psychosocial needs [11]. This lack of competence can manifest in harmful ways such as inappropriate questioning or refusal of care. Surveys across specialties show that clinical experience is often the strongest predictor of provider confidence, yet exposure to TGD patients remains unevenly distributed, and formal training opportunities are sparse [11]. Educational interventions have demonstrated potential to shift knowledge and attitudes but evidence of durable change in practice is limited, especially in rural or resource-poor contexts where providers are isolated from mentorship and peer support [12]. The uneven distribution of trained providers exacerbates geographic disparities, effectively concentrating affirming care in metropolitan areas [9]. The consequence of this is a stark divide between those in urban centers who can obtain timely, competent services and those in rural areas left waiting, traveling, or foregoing care altogether [12, 13].

This review advances the field of transgender health by offering a system-level and cross-domain analysis that is largely missing from existing literature [14, 15]. Whereas most studies examine isolated outcomes — such as mental health, surgical access, STI prevention, or provider training — and those too for urban populations [10], this review integrates these domains to highlight the interdependent and structural forces shaping TGD health across care settings including rural and underserved communities [9]. By highlighting recurring determinants such as regional inequities, stigma, insurance barriers, and gaps in provider preparedness [11], it uncovers cross-domain patterns that individual studies cannot capture and provides a forward-looking map of the structural landscape to reduce disparities in gender-affirming care including GAHT and GAS. A distinctive contribution of this review is the incorporation of artificial intelligence (AI) as an emerging dimension, synthesizing evidence on AI-driven decision support, predictive analytics, natural language processing, virtual training simulations, and voice therapy technologies [1]. In doing so, the paper positions AI as a promising, equity-enhancing frontier with the potential to expand access, strengthen provider competency, reduce administrative burden, and improve continuity of care — particularly in rural and underserved settings [3, 10]. Together, these insights offer a unified foundation for initiatives and future research aimed at advancing global TGD health equity at scale [16].

## Methods

### Search strategy and data sources

A comprehensive literature search was conducted in PubMed to identify studies that evaluate access to, utilization of, or outcomes associated with gender-affirming healthcare among TGD adults. The Boolean search strategy combined terms related to gender-affirming treatments, transgender populations, healthcare access, and disparities: (“hormone therapy” OR “gender affirming hormone therapy” OR “GAHT” OR “gender-affirming surgery” OR “gender confirmation surgery” OR “gender-affirming surgery”) AND (transgender OR “gender minority” OR LGBTQ OR “sexual minority”) AND (rural OR “underserved” OR “medically underserved” OR “health disparities”) AND (access OR barriers OR disparities OR “healthcare delivery”).

### Eligibility criteria

Studies were included if they met all the following criteria: the study was published within the past 10 years through August 2025; the population consisted of transgender or gender-diverse (TGD) adults aged 19 years or older; the study was published in English; and the full text was accessible through institutional or open-access databases. Case reports without analytic data, commentaries, and editorials were excluded.

**Fig. 1:**
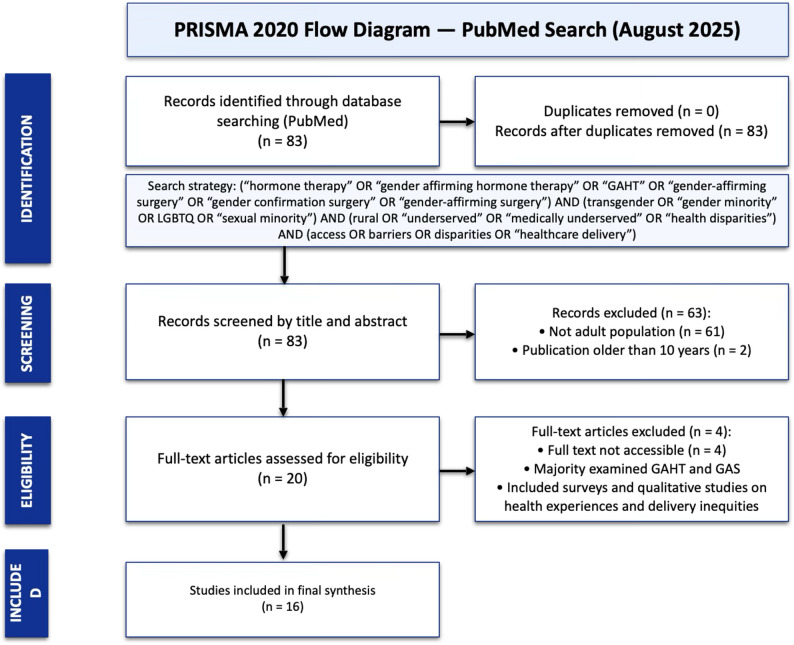
Article selection process

The screening process and reasons for exclusion are summarized in the PRISMA 2020 flow diagram (Fig. 1).

### Data extraction and synthesis

Data from included articles (*n* = 16) were extracted into a standardized template capturing authorship, year, country, study design, population characteristics, intervention or focus (GAHT, GAS, healthcare access), and principal findings. Both quantitative and qualitative data were synthesized to provide a comprehensive and multidimensional understanding of healthcare access, health outcomes, barriers to care, and the emotional impact experienced by TGD individuals.

## Results

Eighty-three articles were identified using the study’s search terms; an additional fifteen articles were identified as cited references. All eighty-three articles were selected by the lead author for initial review. Sixty-three articles were excluded because they did not focus on the adult population or were published before the inclusion time frame. From these, 20 full text articles were assessed for eligibility. Four articles were excluded as the full text was not accessible. Sixteen were utilized for this review; 6 contained findings relevant to Gender-Affirming Hormone Therapy (GAHT), 4 contained findings on Gender-Affirming Surgery (GAS), 1 was focused on retrospective identification and analysis of combined GAHT and GAS utilization, 3 were focused on telehealth intervention for care and 5 explored model of care across various countries (Table 1).

**Table 1:** Key Findings from Recent Studies on Gender-Affirming Care, Social Support, and Clinical Competency for Transgender and Gender-Diverse (TGD) Individuals

Author (Year)	Country	Study Design	Population & Country	Key Findings	Conclusion
Downing J et al. (2024) [19]	USA	Cross-sectional survey	7,740 TGD young adults, USA	In a cohort of 7,740 TGD adults seeking GAHT, only 45% had family support—associated with lower depression (27.4% vs. 33.4%), higher private insurance (44% vs. 36%), and regional differences (more support in Northeast/West; less in South). Support was higher among AFAB than AMAB adults (54% vs. 46%).	Family support is strongly linked to lower depression and more access to gender-affirming care, but support is uneven by region and birth assignment.
Reisner SL et al. (2023) [57]	USA	Longitudinal cohort	6,330 TGD adults, USA	In TGD adults (median age 28; diverse by race/ethnicity; 47% AFAB, 16% nonbinary), 86.3% received GAHT, which significantly lowered anogenital STI risk (aRR 0.75; 95% CI 0.59–0.96) with annual STI positivity of 10–12.5% and notable post-GAHT declines in gonorrhea and chlamydia, especially among trans women, supporting individualized STI prevention.	GAHT reduces gonorrhea and chlamydia rates by 25–35% and enhances sexual health for TGD patients, especially trans women.
Suarez S et al. (2025) [11]	USA	Educational intervention	46 internal medicine residents, USA. (residents (22%) identified as TGD	Training produced lasting gains, with immediate increases in resident comfort and the biggest improvements in PrEP and gender-affirming care knowledge.	Targeted education increases and sustains provider skill and comfort for gender-diverse care, particularly for PrEP and GAHT services.
Marconi M et al. (2025) [24]	Italy	Longitudinal survey (pre/post)	3,102 health professionals, Italy	Among 3,102 Italian clinicians, training produced modest attitude gains, larger improvements in TGD-care skills (2.70 to 3.35; 2.20 to 3.14), and knowledge increases in 8/10 areas (+ 9.9 points in sexual identity accuracy)	Distance learning produces durable improvements in TGD healthcare skills, attitudes, and knowledge, though ongoing refreshers may be needed.
Kilmer LH et al. (2024) [21]	USA	Retrospective matched cohort	3134 patients with gender dysphoria	Gender-affirming plastic surgery was associated with reduced SSRI/SNRI use (–8.8%) and significant declines in depression (–7.7%), anxiety (–1.6%), suicidal ideation (–5.2%) and attempts (–2.3%), and alcohol (–2.1%) and drug misuse (–1.9%).	Mental health and substance use significantly improve following gender-affirming surgery.
Lorello GR et al. (2024) [10]	Canada	Qualitative (hermeneutic)	15 TGD adults, Canada	Following penile-inversion vaginoplasty, TGD individuals shared experiences of body distress, 20-month average waits requiring self-advocacy, anxiety in healthcare settings, self-education via tech, and mixed staff interactions from misgendering to empathy.	Prioritize reduced waits, expanded capacity, centralized referrals, staff training in inclusive language/cultural sensitivity, and holistic care integrating biomedical-psychosocial elements for TGD well-being.
Lane M et al. (2020) [20]	USA	Cross-sectional survey	103 transgender men, USA	In the cohort, 70.3% screened positive for depression and 66.3% for anxiety, despite only 25% and 29.8% having prior diagnoses; scores were significantly higher than cisgender norms (PHQ-9 mean 2.7; GAD-7 mean 2.66; both *p* < 0.0001).	Pre-surgical trans men often suffer severe psychological distress and poor body image, with substantial risk left unmet.
Swonke K et al. (2024) [23]	USA	Cross-sectional survey	53 laryngologists, USA	For most physicians in cohort, TGD care training was mostly experiential (86.8% vs. 20.7% formal), and while 70% felt competent, 72% sought further education and 93% endorsed curricular inclusion, with greatest comfort among those with hands-on experience.	Clinical experience and training are tied to competence; most providers lack both, but there is strong desire for improved education.
Ho TF et al. (2024) [22]	USA	Retrospective EHR cohort	4,587 TGD adults, Utah, USA	In Utah, 65% of TGD adults accessed GAHT or surgery, with rural patients strongly underrepresented (7.3% among those receiving GAHT/GAS), and GAS rates doubled since 2018.	Gender-affirming care expansion has favored urban, White, insured groups, whereas rural TGD adults are twice as likely to be uninsured, and rural TGD people of color face threefold higher uninsured rates than White cisgender residents.
Tabernacki et al. (2025) [18]	USA	Systematic review/meta-analysis	Medical records data of 56,470 transgender men and 41,882 transgender women	TGD adults had substantially higher chronic pain rates (3309–3923 per 100,000 person-years; 1.82–2.08× cis controls), with risk further amplified among TGD women with MDD (HR 1.45) or GAD (HR 1.65).	Gender affirmation provides strong mental health and quality of life improvements for most patients, typically sustained.
Marconi M et al. (2025) [17]	Italy	Cross-sectional survey plus clinical evaluation	959 transgender and gender-diverse adults in Italy ​	More than half reported depression or anxiety; nonbinary adults had poorer health, AFAB individuals had more thyroid disorders, and AMAB individuals had higher osteoporosis and STI rates. Age, education, employment, and GAHT were key predictors of overall health.	TGD individuals in Italy have significant health disparities, underscoring the need for targeted policies, mental health services, and provider training ​
Watson CW et al. (2020) [27]	USA	Qualitative focus groups	37 transgender and gender nonbinary adults, Southern California, USA	PrEP awareness was high, but barriers persisted—discrimination, limited trans-inclusive care, financial strain, side-effect and hormone-interaction concerns, and mental health challenges—while access, prior medication experience, and health motivation facilitated use.	Effective PrEP uptake requires trans-affirming, community-informed strategies that address both structural and psychosocial barriers.
Fisher CB et al. (2018) [25]	USA	Cross-sectional survey	228 transgender youth (14–21 years), USA	Nearly half of youth withheld identity from PCPs over stigma; 25% feared parental confidentiality breaches; only 25% found PCPs helpful, with better information among those disclosing to parents or receiving GAHT.	PCPs should get transgender-inclusive training to address stigma and confidentiality concerns to improve care.
Braun HM et al. (2017) [58]	USA	Cross-sectional survey	87 transgender women in Los Angeles, USA	Among participants, HIV (54%) and feminizing HT (64%) were common, yet only 49% discussed GAHT DDIs; 40% reported ART (12%), HT (12%), or combined (16%) nonadherence due to DDI concerns—indicating major communication and adherence gaps.	Improved communication and coordination around HT–ART interactions are key to supporting adherence in transgender women with HIV.
Douglass KM et al. (2018) [26]	USA	Descriptive observational study	6 TGD patients, 1 trans man and 5 trans women, USA	Homelessness among TGD individuals is driven by family rejection, discrimination, and trauma, compounded by unsafe shelters and high rates of PTSD, depression, and substance use.	Trauma-informed, affirming housing and strong anti-discrimination enforcement are critical to reducing TGD homelessness.
Rhodes SD et al. (2024) [28]	USA	Community-based participatory intervention development	Gay, bisexual, queer, and other men who have sex with men (GBQMSM) and transgender and nonbinary persons, Appalachia, USA ​	A multicultural, peer-navigation and mHealth intervention (Appalachian Access Project) supported HIV/STI/HCV/mpox prevention and GAHT access in rural Appalachia through five modules focused on stigma reduction, service use, communication, and social support.	Training and institutional reforms are necessary to ensure equitable care for LGBTQ+ populations.

### Gender-Affirming Hormone Therapy (GAHT)

Studies on Gender-Affirming Hormone Therapy (GAHT) across rural and urban populations have consistently demonstrated improvements in both mental and physical health among TGD individuals. A clinical qualitative survey of 959 TGD adults from Italy found that over 50% of participants reported depression and anxiety, which improved with GAHT engagement [17]. Evidence from a large U.S. clinical database further highlighted that beyond administration, the timing, duration, and psychosocial context of GAHT critically influence health outcomes, including chronic pain management [18]. Among 56,470 transgender men and women, 40.1% and 50.9%, respectively, were on hormone therapy, with treatment durations ranging from 1 day to over 7,000 days. Overall, 33% of TGD individuals reported adverse healthcare experiences such as treatment refusal and harassment, which correlated with a higher prevalence of chronic pain.

In addition to mental and physical health outcomes, GAHT has been linked to reductions in sexually transmitted infections. A longitudinal study of 6,330 adults in a TGD primary care cohort (median age = 28) found that 86.3% received GAHT and showed reduction in anogenital gonorrhea and chlamydia infections, especially among transgender women [5].

Social and structural factors also shape access to GAHT and related outcomes. A U.S.-based study showed that among 7,740 TGD adults between ages 18–25, patients with family support reported higher rates of private insurance (44% vs. 36%, *p* < 0.001) and lower rates of being uninsured (29% vs. 34%, *p* < 0.001) compared with those without family support [19]. This translated to TGD patients with family support having lower rates of depression and higher rates of accessing fee-for-service GAHT and GAS, leading to their preferred name aligning with legal name, living as their gender full time, and expressing their gender for 2 + years compared with those without family support [19].

These findings demonstrate that GAHT contributes to meaningful improvements in well-being, yet the extent of its benefits depends on consistent treatment and the broader social environment [19]. Factors such as discrimination, negative healthcare interactions, insurance availability, and family support substantially influence how GAHT is experienced and how effectively it improves health outcomes. Overall, improving outcomes with GAHT depends on expanding access and addressing the social and structural barriers that affect how care is delivered and experienced [5, 18].

### Gender-Affirming Surgery (GAS)

The studies on GAS consistently demonstrate measurable improvements in mental health outcomes among TGD individuals. However, limited access, due to structural, logistical, and psychosocial barriers, or prolonged delays in receiving these procedures led to marked psychological distress among the cohorts. In cases where surgery is unavailable or deferred, the rates of depression, anxiety, and suicidality increase.

Qualitative data further highlight persistent systemic barriers to care. In a study by Lorello et al. 15 transgender patients who underwent penile inversion vaginoplasty at a Toronto hospital were interviewed. Participants described ongoing body distress, an average 20‑month surgical wait requiring active self‑advocacy, and heightened anxiety in healthcare settings [10]. Many described relying on self‑education through technology and encountered a spectrum of staff interactions, from misgendering to empathy.

Pre-surgical populations remain particularly vulnerable. In one study of transgender men, over 70% screened positive for depression and 66% for anxiety, rates far exceeding those among cisgender peers [20]. Encouragingly, a cohort study of 3,134 patients found that gender-affirming surgery was associated with significant improvements in mental health outcomes including decreases in depression (–7.7%), suicidal ideation (–5.2%), suicide attempts (–2.3%), and substance misuse [21]. In the same cohort, the use of antidepressant medications declined by 8.8% postoperatively, indicating sustained mental health benefits.

While more people are getting access to care than before, not everyone is benefiting equally, and clear gaps remain depending on factors such as where someone lives or their access to resources. In Utah, rates of gender-affirming surgery have doubled since 2018, yet access remains skewed toward urban populations, with rural individuals significantly underrepresented [22].

Overall, evidence suggests that while GAS is highly effective in improving mental health, timely and equitable access remains a major challenge. Long wait times, financial barriers, and geographic disparities lead to delayed care, and those delays are closely tied to ongoing or worsening psychological distress. Even as access has expanded, disparities persist, with rural and under-resourced populations continuing to face the greatest challenges. Ultimately, the benefits of surgery are well documented and show the urgent need to expand access and reduce barriers to timely care as critical next steps.

### Healthcare provider training and competency in TGD care

Studies show provider training in TGD care remains limited, yet even modest educational interventions are associated with meaningful improvements in clinical confidence, knowledge, and quality of care. An educational intervention among internal medicine residents demonstrated that targeted training led to sustained improvements in knowledge, skills, and attitudes, with most participants reporting comfort in providing TGD care post-training [11]. The largest gains were observed in PrEP knowledge and gender-affirming care delivery. Distance learning programs for healthcare providers have been shown to produce durable improvements in TGD care competencies. In a longitudinal study of 3,102 Italian clinicians, Marconi et al. (2025) found modest but significant attitude gains at six months, alongside larger improvements in skills (2.70→3.35; 2.20→3.14) and knowledge across most domains, including a ~ 10-point increase in sexual identity accuracy, suggesting refresher training may be needed to sustain attitudinal change [17].

Provider competency for GAS care is also strongly tied to experience. 93% of laryngologists endorsed trans health curriculum, though 79.3% had never received formal trans health training and claimed inexperience treating TGD patients [23]. 86.8% reported learning TGD care through informal, experiential means rather than formal training, and although 70% felt competent, 72% still desired additional education [23]. Lack of provider competency directly affects patient care: nearly half of transgender youth reported withholding their gender identity from primary care providers due to anticipated stigma, and only 25% found providers helpful in sexual health discussions [25].

Clinician approach is especially important in the context of gender-affirming surgery, where mental health and medical care are closely intertwined. Douglass et al. calls for trauma-informed, trans-competent psychiatric care that goes beyond standard mental health approaches and considers the interactions between hormone therapy and psychiatric medications [26]. The authors highlight how clinicians must actively address minority stress, including experiences of stigma, discrimination, and social marginalization that shape mental health outcomes in TGD populations. They further emphasize the need for greater clinical awareness of how gender-affirming hormone therapy may interact with psychiatric medications, underscoring the importance of integrated, multidisciplinary care rather than treating mental health and gender-affirming treatment as separate domains.

The studies highlight a clear gap between the need for TGD-competent care and the level of training most providers receive. While even brief training improves knowledge, confidence, and skills, it is not consistently implemented and does not always translate into practice. As a result, many clinicians rely on informal experience, and gaps in competency can lead to patient mistrust, nondisclosure, and reduced engagement in care. These challenges are especially important in the context of gender-affirming surgery, where mental health, hormone therapy, and surgical care are closely connected. Overall, the literature highlights the need for more consistent, integrated training approaches that better prepare clinicians to meet the needs of TGD patients [27].

### Policy and geographic determinants of TGD healthcare access

Policy, structural, and geographic factors continue to influence care accessibility for TGD individuals, with rural communities experiencing the greatest disparities stemming from workforce limitations and uneven resource distribution.

At the policy level, structural factors continue to shape access to care. Data shows that discrimination, financial strain, and lack of trans-inclusive healthcare environments significantly hinder access to preventive services such as PrEP [27]. Housing instability further compounds these challenges. Among TGD individuals experiencing homelessness, barriers to care were driven by family rejection, systemic discrimination, and unsafe shelter environments, all of which intersect with healthcare access and outcomes [26].

A community-based intervention in rural Appalachia demonstrated how targeted, culturally tailored strategies can help bridge existing gaps in care for TGD individuals. The Appalachian Access Project implemented a culturally tailored, peer-navigation and mHealth approach to support HIV/STI prevention and access to gender-affirming care, including GAHT [28]. The intervention focused on reducing stigma, improving communication, and increasing service uptake. Peer navigators played a central role in building trust and connecting individuals to care in areas with limited provider availability.

Geographic location is another key determinant of access to care for the TGD population, and these inequities are especially pronounced in rural settings. In Utah, rural TGD individuals were significantly underrepresented among those receiving GAHT or surgery and were more likely to be uninsured [22]. Similar regional patterns emerged nationally: family support for GAHT was more common in the Northeast and West than in the South, correlating with higher insurance coverage and better mental health outcomes [19]. These findings suggest that sociopolitical climates influence both support systems and healthcare access.

Taken together, these data reveal that both place and policy play a major role in shaping access to care. Moving forward, progress will depend on addressing underlying structural challenges and strengthening community-based, culturally tailored approaches.

## Discussion

This review synthesizes the last 10 years of research describing healthcare access, delivery, and outcomes among TGD populations across multiple countries and care settings. Collectively, these findings show how familiar challenges in rural and underserved healthcare — resource distribution, provider training, and psychosocial context — also shape access to and effectiveness of gender-affirming care. The emerging evidence emphasizes variability more than uniformity in the healthcare experiences of TGD communities.

### Access and contextual factors

Disparities in access remain a central finding [4, 7, 9]. This inequity is shaped by a complex interplay of factors beyond availability of service alone. Insurance, geography, and provider availability lead to limited access that directly affects health, often delaying care and worsening outcomes.

Studies from the U.S., Canada, and Italy show that rural, uninsured, and minority TGD individuals face lower rates of GAHT and surgery and longer wait times [4, 10, 22, 24, 29]. Importantly, having services available does not always mean they are accessible. A rural Appalachia program showed that stigma and system navigation barriers still limited care, even when services existed, and that peer-supported, community-based approaches could help bridge these gaps [28].

Addressing these disparities will require changes across both policy and care delivery. Reducing insurance barriers can expand access to gender-affirming services, while hybrid telehealth models offer a practical way to reach more patients without sacrificing quality [30]. Evidence shows that eliminating insurance exclusions and prior-authorization and regulatory barriers (including testosterone restrictions) and enforcing consistent implementation can ensure reliable access to gender-affirming care [31]. Hybrid care models that combine in-person and telehealth visits have been associated with improved access for rural and low-income populations while maintaining care quality and satisfaction [30, 32], while mandatory, community-informed LGBTQ+-affirming training can reduce bias and strengthen provider competency [33, 34]. Integrated care models that co-locate primary care, hormone therapy, mental health services, surgical consultation, and social work help reduce fragmentation and support navigation of complex systems [35, 36], complemented by peer navigation approaches that leverage community knowledge to improve access, mitigate stigma, and sustain care continuity, particularly in underserved settings [37, 38].

### Social and family support

Several large-scale surveys highlight the influence of family and community environments on patient engagement in gender-affirming care, and the corresponding mental-health outcomes [8]. This matters because support systems can either reinforce or undermine access to care. Supportive environments are linked to better mental health, greater trust in healthcare systems, and more sustained engagement with gender-affirming services, while lack of support can contribute to disengagement and poorer outcomes.

A consistent pattern emerges across the literature. In a large U.S. cohort of 7,740 TGD young adults, individuals with family support had lower rates of depression and were more likely to have private insurance, which facilitated access to GAHT and related services [19]. Regional differences emerged, with higher levels of family support reported in the Northeast and West compared to the South, suggesting that broader social environments shape both support systems and access to care. Studies of housing instability showcase the consequences of lacking support, where family rejection contributes to homelessness and further limits access to healthcare [26].

Moving forward, improving TGD health outcomes will require strengthening family and community-based interventions that improve mental health and sustain care engagement. Evidence shows that integrating family-based affirming care within clinical settings including psychoeducation, caregiver coaching, and structured space to process responses to gender identity serves to reduce TGD youth suicide risk [39]. Complementary approaches such as parent peer-mentorship programs can build caregiver confidence and long-term support capacity [40]. Programs led by TGD community members that connect parents with peer mentors have shown promising benefits in healthcare navigation, emotional support, and linkage to services, especially for those facing intersecting barriers [38]. Expanding structured gender-affirming family therapy and multi-family support groups strengthens relational support and mitigates isolation [39, 41]. Targeted efforts addressing housing instability among youth rejected by families further prevent disengagement from care [19]. Together, these approaches point to a cohesive, evidence-based approach for building protective support systems that improve outcomes and continuity of care [19, 39].

### Clinical outcomes and mental health

Across the studies, clinical and public health outcomes point toward improved well-being following gender-affirming interventions. This is an important consideration in the well-being of TGD individuals because mental health disparities remain a central concern in this population. These results add to a growing body of longitudinal evidence that suggests individualized, affirming healthcare can yield mental and physical health benefits [10]. Improvements in depression, anxiety, and related outcomes suggest that access to affirming care is closely tied to overall health and quality of life.

Across studies, GAHT was linked to reductions in sexually transmitted infections, particularly gonorrhea and chlamydia, alongside broader improvements in engagement with care [5]. Surgical interventions showed similarly strong effects, with decreases in depression, suicidal ideation, and substance use following gender-affirming procedures [21]. At the same time, pre-surgical populations remained highly vulnerable, with elevated rates of depression and anxiety observed prior to intervention [20]. Additional findings suggest that outcomes are shaped by factors such as timing and continuity of care, as well as broader healthcare experiences, including discrimination and access barriers [18].

The value of gender-affirming care lies in shaping both mental and physical health, including reduced depression, dysphoria, and suicidality [42, 43]. Future efforts should focus on ensuring timely access to care and integrating behavioral health support, particularly for individuals early in transition or awaiting treatment. Early access, especially for adolescents, is associated with substantial reductions in depression and self-harm, while those waiting for care remain highly vulnerable [43]. Integrating behavioral health into affirming care settings further improves outcomes by reducing fragmentation through mechanisms such as screening, warm handoffs, and same-day support [44–46]. For individuals awaiting care, particularly surgical care with extended delays, bridge programs offering counseling, peer support, and harm reduction can mitigate worsening mental health during the long wait periods [47]. Multidisciplinary clinic models that co-locate medical, surgical, and behavioral services are shown to support continuity and individualized care planning [43, 48]. Taken together, these measures reinforce the idea that access to gender-affirming interventions is associated with meaningful reductions in psychological distress and suicidal ideation.

### Provider preparedness and training

Provider preparedness emerged as an area of both challenge and opportunity [6]. Surveys and qualitative studies demonstrate that many clinicians begin with limited training in transgender health, leaving gaps in confidence, knowledge, and clinical decision-making [6]. Provider competency directly shapes patient experiences. Limited training can contribute to discomfort in care delivery, inconsistent practices, and reduced patient trust, while well-trained providers are better equipped to offer affirming, effective care [11, 12].

Findings from the literature reveal a persistent gap in provider preparedness. Most providers reported relying on informal, experience-based learning rather than formal training, with many expressing a desire for additional education [23]. Structured training programs demonstrated clear benefits. An educational intervention among internal medicine residents led to sustained improvements in knowledge, skills, and confidence, with over 90% reporting comfort in providing TGD care [11]. A large-scale training initiative in Italy showed measurable gains in clinical skills and knowledge, particularly in areas such as hormone therapy and gender identity, although changes in attitudes and clinical practice were more modest [24].

There’s a clear need to strengthen how TGD care is taught and need for scalable training within existing medical-education frameworks [12]. This requires shifting from fragmented, self-directed learning to standardized, competency-based education embedded across training and practice. The World Professional Association for Transgender Health (WPATH) offers evidence-based guidance on hormone therapy, preventive care, surgical aftercare, and culturally competent communication, giving providers a standardized framework to deliver safe, affirming, and consistent care for TGD patients. Integrating this content into required curricula can strengthen clinical competence, reduces practice variability, and improves patient outcomes [33, 49]. Ongoing continuing education tied to credentialing can ensure physician alignment with evolving care standards [49]. Scalable, telehealth-enabled training models further extend reach to underserved settings [50], and pairing these with quality metrics and community-informed curriculum design is shown to support continuous improvements in patient care outcomes [34]. Collectively, these approaches provide a consistent, accountable model of provider training that can improve TGD care quality and patient trust.

### Intersecting challenges and emerging approaches

Building on the access findings above, persistent gaps remain most apparent among rural and uninsured TGD populations [4, 7]. Intersecting factors, such as housing instability, unemployment, and discrimination, limit both access and continuity of care, reinforcing one another and contributing to poorer mental health [26]. These overlapping barriers emphasize the need for integrated interventions that address structural barriers and social determinants simultaneously. Expanding comprehensive coverage for GAHT and surgery, while reducing cost barriers and easing prior authorization across Medicaid and private insurance, can help close persistent gaps in access to care [51]. Hub-and-spoke telehealth models that connect local clinics with specialized gender centers have increased GAHT uptake by 45% in rural Appalachia, highlighting a practical and scalable approach to expanding access to care [30, 52]. Expanding peer navigation programs led by TGD community members is another practical approach to improving insurance enrollment and medication access, helping reduce delays that affect a majority of rural patients [38]. Housing-first initiatives paired with healthcare navigation support in shelters and transitional housing are associated with up to fourfold improvements in access to gender-affirming care for LGBTQ+ youth [26, 51]. Mobile health units can further extend services to remote communities. Culturally tailored outreach via local centers, faith-based organizations, and social media are shown to play a key role in countering stigma in isolated populations [52]. The evidence-based, community-driven strategies provide promising outlook for addressing intersecting structural barriers and creating sustainable pathways for equitable access to gender-affirming care.

### Reimagining gender-affirming care through artificial intelligence

Artificial intelligence (AI) is emerging as a new frontier within gender-affirming healthcare showing tremendous potential to address persistent gaps in access, quality, and patient experience. Limited yet successful AI-enhanced techniques on clinical decision support systems (CDSS) are showing promise to guide hormone therapy dosing, laboratory monitoring, and interaction screening, in rural and geographically dispersed settings with limited specialist availability. Explainable AI (XAI) models that are geared towards driving greater transparency with their operation and allowing users to understand how they reach their conclusions provide clinicians with recommendations along with interpretable rationales, and are aimed at improving safety, confidence, and standardization of care across diverse practice environments such as rural and underserved communities [14, 16].

The AI driven data science domain - Predictive analytics - that has reached maturity in technology is now appearing in healthcare and showing initial promise to identify TGD individuals at elevated risk of mental-health crises. Machine-learning models - such as the ones used for prediction - are trained on longitudinal clinical data, patient-reported outcomes, and passive digital signals can assist in mitigating psychological distress during early transition or pre-surgical periods and allow timely, human-mediated outreach from behavioral-health specialists or peer navigators [15].

Another set of AI applications - Natural language processing (NLP) - that rely on human speech and text for computations offers unique opportunities to streamline administrative tasks and improve care equity. NLP tools can identify transgender patients within electronic health records, identify and correct misgendering, automate letters of medical necessity for surgical referrals, and identify patients who may benefit from targeted outreach or preventive care [53, 54]. These NLP based AI tools reduce documentation burdens on an overly fatigued healthcare system and greatly improve both patient experience and care continuity.

AI-driven training simulations can enhance provider preparedness. Using virtual patient simulations that rely on conversational AI models can empower clinicians to practice gender-affirming communication and receive automated real-time feedback on language and scenario handling to ensure consistent, repeatable skill development that complements traditional clinical education [55].

Finally, AI-supported voice and behavioral therapy platforms are showing great promise in gender-affirming voice modification where acoustic analysis provides patients with real-time feedback during specialized speech therapy, increasing access where specialized speech therapy is unavailable [53, 55].

Table 2 summarizes AI applications in transgender and gender-diverse (TGD) care by domain, core function, and specific uses.

**Table 2:** Emerging AI Domains Supporting Gender-Affirming Hormone Therapy (GAHT) and Gender-Affirming Surgery (GAS)

AI Domain	Core Function	Applications in TGD care
Clinical Decision Support Systems (CDSS) and Explainable AI (XAI)	Deliver explainable and well-structured clinical guidance for gender-affirming care.	GAHT dosing recommendations, monitoring labs for estrogen/testosterone therapy, screening for drug–drug interaction in GAHT medications (e.g., estrogen, testosterone, spironolactone) and peri-operative drugs (anesthetics, anticoagulants, analgesics) [59].
Predictive Analytics	Used to identify early biological, psychosocial, or surgical risk signals.	Detect mental-health risk during early GAHT transition periods, predict psychosocial stress surrounding GAS planning, identify factors associated with delayed recovery or postoperative complications [60–62].
Natural Language Processing (NLP / EHR Automation)	Helps drive documentation accuracy and improve administrative workflows.	Identify TGD individuals across fragmented health systems, correct misgendering in GAHT/GAS charts, automate documentation and forms of medical necessity for GAS (vaginoplasty, phalloplasty, or facial feminization surgery), identify gaps in GAHT follow-up labs [63–65].
AI-Based Training Simulations	Build healthcare provider expertise in gender-affirming clinical skills.	Provide simulation scenarios for GAHT counseling, simulations for peri-operative GAS communication and trauma-informed care, feedback on affirming language for provider training [66].
Voice & Behavioral Therapy AI	Expand access to gender-affirming voice training and behavioral support.	Real-time acoustic analysis for transfeminine/transmasculine voice training as a part of GAHT care, remote therapy for patients preparing for or recovering from GAS, voice tools to support gender presentation and identity congruence alongside GAHT [55, 67].

### Broader implications

Viewed collectively, gender-affirming healthcare represents one timely example within the wider landscape of rural and underserved healthcare. The evidence highlights that outcomes improve when care is accessible, affirming, and integrated across disciplines — principles that are broadly applicable beyond any single population [12]. As the evidence expands, including with the emergence of AI, careful attention to bias mitigation [14], patient privacy, and human oversight [56] is essential to ensure all approaches serve as an equity-enhancing tool rather than exacerbating disparities. Continued emphasis on training, accessibility, and longitudinal evaluation may yield insights broadly applicable across underserved healthcare settings, while improving the experiences of TGD individuals navigating specialized or identity-related care.

## Conclusions

The evidence synthesized here highlights that the healthcare challenges faced by TGD individuals reflect a broader pattern shared across many underserved communities. Barriers such as fragmented insurance coverage, limited provider availability, and inconsistent access to timely care, continue to limit equitable access. By examining these issues through the lens of TGD health, these challenges reveal systemic weaknesses that transcend any one population and also impact rural and low-income communities globally. Looking ahead, innovative approaches - such as competency-based provider education, telehealth, and community-driven outreach, offer promising, scalable solutions. Emerging evidence in use as a support tool within gender-affirming healthcare warrants evaluation, responsible implementation, clinician oversight, and continuous evaluation of outcomes. Applications of artificial intelligence further reinforce this trajectory. Across decision support, risk prediction, documentation streamlining, and virtual training, early evidence suggests that AI can help reduce administrative burden, improve diagnostic consistency, and extend specialist-level guidance into rural and low-resource environments. While still in early stages, these technologies, when paired with ethical safeguards and human oversight, represent a practical pathway to more consistent, accessible, and patient-centered gender-affirming care. Efforts to advance TGD health can serve as a catalyst for strengthening care systems for all populations facing barriers. Moving forward, progress in TGD healthcare can drive a wider transformation toward more resilient, equitable, and person-centered healthcare systems that serve all individuals facing barriers to care.

## Data Availability

No datasets were generated or analysed during the current study.

## Abbreviations

AFAB: Assigned Female at Birth
AMAB: Assigned Male at Birth
AI: Artificial Intelligence
ART: Antiretroviral Therapy
CDSS: Clinical Decision Support System
GAHT: Gender Affirming Hormone Therapy
GAS: Gender Affirming Surgery
GAD: Generalized Anxiety Disorder
GBQMSM: Gay, Bisexual, Queer Men and Men who have Sex with Men
GSD: Gender and Sexual Diversity
GSM: Gender or Sexual Minority
DDI: Drug-Drug Interactions
HCV: Hepatitis C Virus
HIV: Human Immunodeficiency Virus
HT: Hormone Therapy
MDD: Major Depressive Disorder
Mpox: Monkeypox
NLP: Natural Language Processing
PCP: Primary Care Provider
PrEP: Pre-exposure prophylaxis
SGM: Sexual and Gender Minority
STD: Sexually Transmitted Disease
STI: Sexually Transmitted Infections
TGD: Transgender and Gender Diverse
XAI: Explainable AI
